# Ventriculostomy and Infection: A 4-year-review in a local hospital

**DOI:** 10.4103/2152-7806.69033

**Published:** 2010-09-09

**Authors:** TS Tse, KF Cheng, KS Wong, KY Pang, CK Wong

**Affiliations:** Department of Neurosurgery, Pamela Youde Nethersole Eastern Hospital, 3 Lok Man Road, Chai Wan, Hong Kong SAR

**Keywords:** External ventricular drain, ventriculostomy-related infections, ventriculitis prevention measures

## Abstract

**Background:**

To review the complication rate of ventriculostomy-related infection in a local regional hospital, to identify risk factors of infections and suggest measures to prevent infections.

**Methods:**

Retrospective review of all cases involving ventriculostomy in one centre of a 4-year-period (Pamela Youde Nethersole Eastern Hospital, a local regional hospital in Hong Kong). 336 cases of admission involving 328 patients with a total of 368 ventriculostomy performed in the centre in a 4-year-period. Main outcome measures include Rate of infection and risk factors related to infections.

**Results:**

10 cases of out 336 cases (2.98%) of ventriculostomy were complicated by infection. Neither the duration of ventriculostomy, revision, urokinase instillation or haemorrhage showed significance in the rate of ventriculostomy-related infection. The low infection rate is compatible with other international literatures that used strict infection control measures.

**Conclusion:**

Strict measures for prevention aid in achieving a low complication rate of ventriculostomy related infection.

## INTRODUCTION

Indwelling ventriculostomy catheter or External Ventricular Drain (EVD) is a common Neurosurgical procedure. It can be defined as a surgical establishment of a communication between cerebral ventricular system and the external environment (outside of the human body). EVD is integral in the management of patients with a variety of neurosurgical conditions, including elevated intracranial pressure (ICP), a variety of intracranial haemorrhages, intracranial tumour, traumatic head injury, cerebral oedema and ICP monitoring. However, ventriculostomy, as with all surgical procedures, sustains a risk for complications. One of the complications of concern is ventriculostomy related infection (VRI). The reported incidence of ventriculostomy related infection varies from 0% to 22%.[11] A number of risk factors of ventriculostomy related infection had been identified but the significance of these factors varied among various studies.[2,7,8,11,12,14] Various prevention protocols and practice guidelines had also been suggested with an aim to reduce the incidence of VRI.[3] A previous 5-year review of ventriculostomy at the author’s centre, the infection rate of EVD is <1%.[1]

This is a 4-year retrospective review of all the consecutive cases requiring ventriculostomy at the author’s centre (a local hospital in Hong Kong) in which the complication of ventriculostomy related infection addressed.

## METHODS

A retrospective review of the records of all the patients with ventriculostomy performed at the Pamela Youde Nethersole Eastern Hospital in Hong Kong SAR during the study period. The study period is from 1^st^ of July 2005 to 30^th^ of June 2009.

The Clinical Data Analysis and Reporting System (CDARS) were utilized for case searching. Search requirements on CDARS included Procedure date between 1^st^ July 2005 and 30^th^ of June 2009 and all admission episodes with procedure code of Ventriculostomy, Insertion of Catheter for drainage of cerebrospinal fluid (CSF), Operation to establish drainage of ventricle and/or Twist drill hole for external ventricular drainage. Exclusion criteria: All cases with intracranial pressure monitoring not using external ventricular drain. (eg. Subdural catheter, Lumbar drain.)

Patients’ data were retrieved from the Electronic patient record (EPR) and Clinical Management System (CMS). Data retrieved through medical records includes patient demographic data (age, sex), presenting diagnosis, Indication of ventriculostomy, duration of ventriculostomy and suspected risk factors. Laboratory investigation results reviewed include microbiological culture of Cerebrospinal fluid (CSF), EVD tip, blood, urine and sputum and biochemistry and microscopy of CSF. Outcome measured includes complication of infection, treatment and outcome of cases with ventriculostomy related infection

Data Analysis was performed with Microsoft Office Excel 2007 and GraphPad Software QuickCalcs.

Outcome measure includes duration of ventriculostomy, revision rate, CSF or EVD culture positive rate, rate of ventriculostomy related infection, Outcome of cases with ventriculostomy related infection

The results were compared with published international studies.

## RESULTS

336 cases of admission involving 328 patients with a total of 368 external ventricular drains performed at the author’s centre during the period of study.

All ventriculostomy were performed in the operation theatre. The insertions were performed in the operating theatre with the aim to decrease infection rate and improvement of surgical safety.

None the ventriculostomy catheters were antibiotics coated.

Peri-operative prophylactic antibiotics were routinely administered to all patients. For patients with the ventriculostomy procedure within 1 week of admission to hospital, a one-gram intravenous dose of Ceftriaxone was administrated at the start of the operation and every 8 hours for three doses after the operation. For patients with hospital stay of more than 1 week before the operation, peri-operative antibiotic prophylaxis involved routine administration of one-gram intravenous dose of vancomycin and one-gram dose of sulbactam/cefoperazone once before the operation and every 8 hours for three doses after the operation.

All patients were nursed in the intensive care unit (ICU), high dependent unit (HDU) or Neurosurgical ward after the insertion of the EVD until the ventriculostomy had been removed. Continuous ICP monitoring with an external transducer was deployed for all cases until the ventriculostomy was removed. CSF samplings were only performed when indicated and performed with aseptic technique. The EVDs were removed once it had served its clinical purpose and the removal of catheter involved aseptic technique. The tips of the ventriculostomy catheters were routinely saved for bacterial culture upon removal, with exceptions only with cases of patients who succumbed to medical conditions unrelated with ventriculostomy related infection before the ventriculostomy catheters were removed.

### Gender

**Figure 1:**
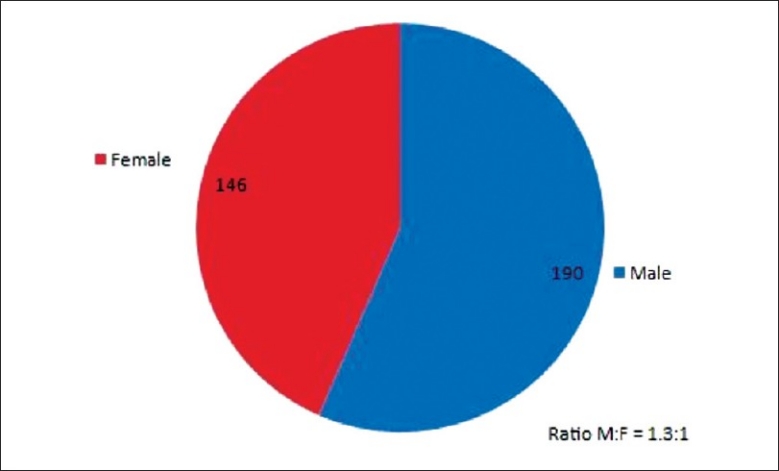
Gender distribution

The study revealed a higher number of male patients (190 cases) compared to female patients (145) with a male to female ratio of 1.3 to 1. [Figure 1, Table 1.]

**Table 1:** Gender

Gender	No. of Case
Male	190
Female	146
Male: Female = 1.3:1	

### Age (years)

**Figure 2:**
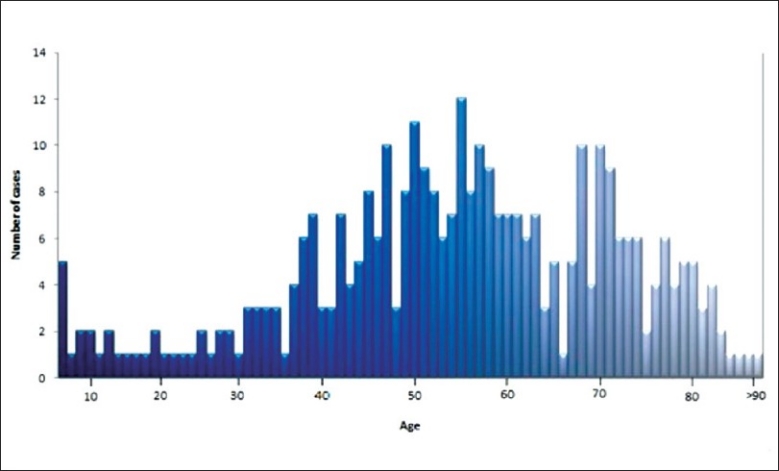
Age distribution continuous

**Figure 3:**
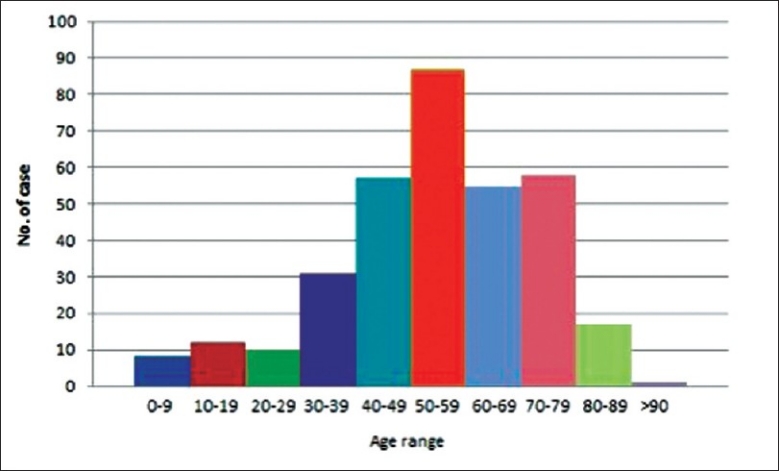
Age distribution by group

The mean age of all the patients was 54.1 years old with a standard deviation (SD) of ± 18.01. The mode and median of the all the patients’ age were all 55 years old. The oldest patient was 98 years on day of operation. There were 4 patients who underwent EVD inserted within the 1 year of birth and the youngest only 1 day old. Most number of patients had the operation during the age range 50-59 years old. A large majority of the total number of patients had the ventriculostomy during the age range of 40-79 years old. [Figure 2 and 3, Table 2].

**Table 2:** Age

Age	Years
Mean	54.1
Mode	55
Median	55
Oldest	98
Youngest	1 day

### Indications of ventriculostomy

**Figure 4:**
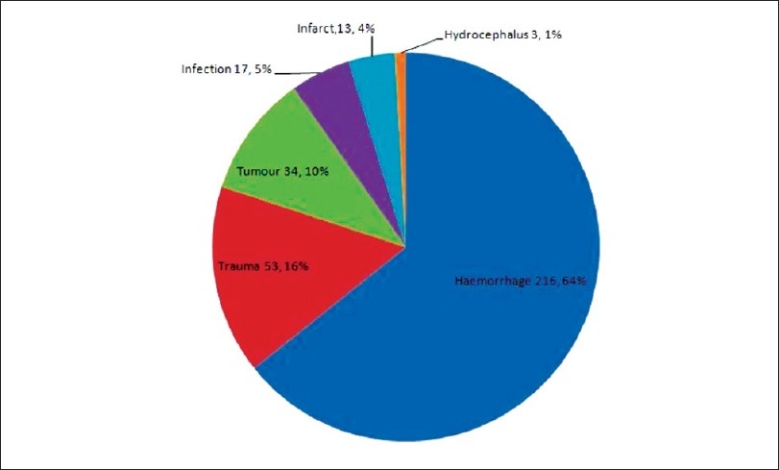
Indication of ventriculostomy

The indication of ventriculstomy for a majority of the patients (64.3%, 216 cases) was haemorrhage which included cases of subarachnoid haemorrhage (SAH), intraventricular haemorrhage (IVH), intracerebral hemorrhage (ICH) and cerebellar haemorrhage. The second most common indication was trauma (15.8%, 53 cases) and it includes traumatic fracture of the skull requiring craniotomy or craniectomy, traumatic acute epidural haemorrhage requiring clot evacuation and traumatic head injury requiring ICP monitoring. The indication of tumour(10.1%, 34 cases) involved cases of tumour related obstructive hydrocephalus or operative removal of intracranial tumour with ventriculostomy as post-operative ICP monitoring. Indication of infection represent cases of infected CSF shunt system, tuberculosis meningitis induced hydrocephalus and infantile ventriculitis for intraventricular antibiotics irrigation and CSF sampling. Infarct as an indication (3.9%, 13 cases) involves cases with cerebellar infraction related hydrocephalus and post infarction cerebral oedema with significant massive effect. [Figure 4. Table 3].

**Table 3:** Indications for EVD

Indications for EVD	No. of cases	Percentage of total
Hemorrhage	216	64.3
Trauma	53	15.8
Tumor	34	10.1
Infection	17	5.1
Infarct	13	3.9
Hydrocephalus (causes other than any of the above mentioned)	3	0.9

### Duration of ventriculostomy

**Figure 5:**
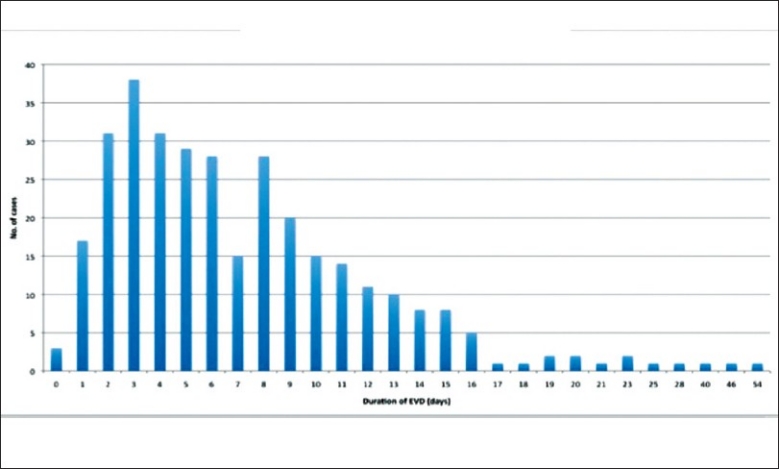
Duration of ventriculostomy by episode

**Figure 6:**
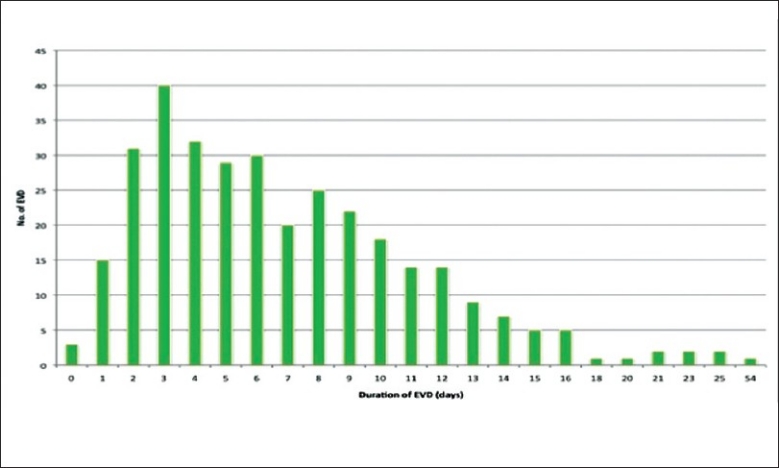
Duration of ventriculostomy per catheter

The duration of ventriculostomy in this review was regarded as the number of days from the operative insertion of EVD to the day of removal of ventriculostomy. As some cases involved revision of ventriculostomy or more than one ventriculostomy catheter inserted in patients during the same episode, while others require re-insertion of ventriculostomy shortly after removal of ventriculostomy catheters. Thus the duration of ventriulostomy were measured on 2 separate values: the duration of each EVD catheter (duration per EVD) and the total number of days in which at least 1 ventriculostomy catheter was present on the patient in the episode of admission (duration per episode). Results summarized by table and chart below. [Figure 5 And 6. Table 4].

**Table 4:** Duration of EVD

Duration of EVD	Days
Mean (per episode)	7.39 (SD=6.10)
Mean (per EVD)	6.93 (SD=5.11)
Mode (per EVD and per episode)	3
Median (per EVD and per episode)	6
Longest	54
Shortest	0

### Manipulation

**Figure 7:**
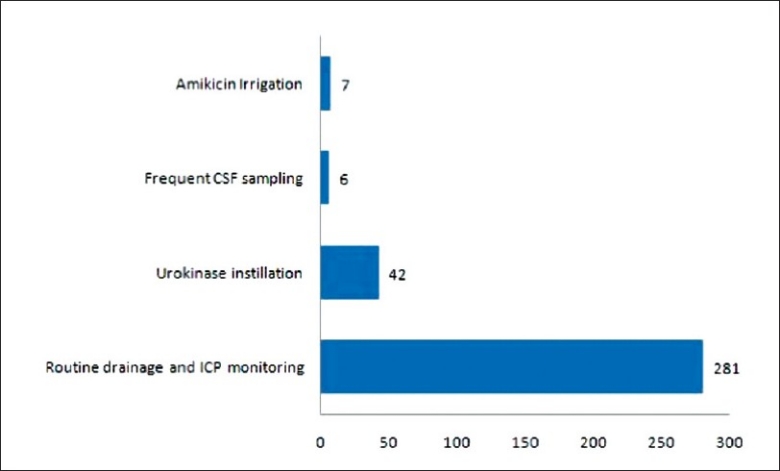
Manipulation of ventriclostomy

During the period in which the external ventricular drain was inserted, a number of cases involved manipulation of the external system. Such manipulations were performed under aseptic technique and only performed if clinically indicated. However, the data collected in this study only involved 3 types of manipulations (urokinase instillation, amikicin irrigation and frequent CSF sampling). Urokinase instillation via the ventriculostomy was performed for patients with intraventricular haemorrhage (IVH) after aneurismal rupture and arteriovenous malformation were ruled out by CT cerebral angiogram or Digital subtraction cerebral arteriogram.[10] Amikicin irrigation were used in patients with central nervous system (CNS) infections which include pre-existing meningitis, post traumatic and post-operative CNS infections in the presence of positive bacterial culture in CSF via a ventriculostomy in situ.[15] CSF sampling were performed in cases with fever, raised serum white cell count, impaired mental status, duration of EVD longer than 5 days and manipulations which involved exposing the closed sterile external collection system for drained CSF. Frequent CSF sampling, which we defined as repeated CSF sampling of once every 2 to 3 days for more than 3 occasions, only involved patients with pre-existing meningitis or suspected post operative CNS infections of which frequent CSF sampling via the ventriculostomy was performed to monitor the response to treatment of the respective conditions. The table and graph represents the number of patients with urokinase instillation, amikicin irrigation and frequent sampling of CSF (without any of the 2 former procedures involved). [Figure 7, Table 5].

**Table 5:** Manipulation

Manipulation	No. of case	Percentage
Nil	281	83.6
Urokinase instillation	42	12.5
Amikicin irrigation	7	2.0
Frequent CSF sampling	6	1.8

### Revision of ventriculostomy

**Figure 8:**
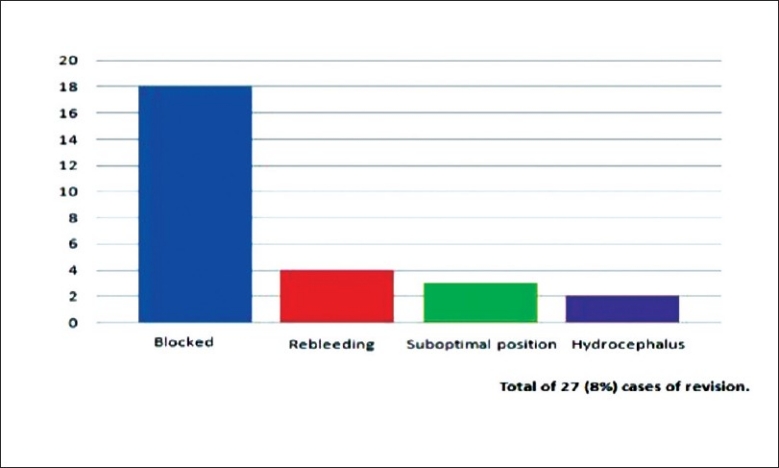
Indication for revision of ventriculostomy

**Figure 9:**
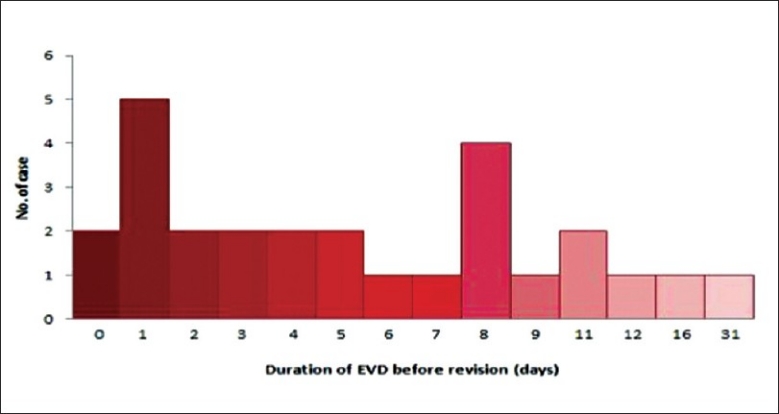
Duration of ventriculostomy before revision

A small number of cases required revision of ventriculostomy. A majority of these cases was due to blockage of the ventriculostomy diagnosed by observation of CSF flow and CT imaging. Other indications for revision include re-bleeding from previous intracranial haemorrhage, suboptimal position of the ventriculostomy and hydrocephalus (not related to the above mentioned causes). Most of the revisions were performed within 8 days of the initial insertion of ventriculosotmy with a mean duration of ventriculostomy before revision at 6.2 days. Most of the cases with a revised ventriculostomy required a longer duration before removal of the ventriculostomy. [Figure 8 and 9. Table 6, 7 and 8].

**Table 6:** Indication for revision

Indication for Revision	No. of cases	Percentage
Total no. of revised cases	27	8
Blockage of EVD	18	5.3
Bleeding	4	1.1
Suboptimal positioning of EVD	3	0.9
Hydrocephalus	2	0.6

**Table 7:** Duration of EVD before Revision

Duration of EVD before Revision	Days
Mean	6.2
Median	5
Shortest	0
Longest	31

**Table 8:** Total Duration of EVD in revision cases

Total Duration of EVD in revision cases	Days
Mean	15.3
Median	14
Shortest	4
Longest	46

### Infection

#### Culture of the ventriculostomy tip

**Figure 10:**
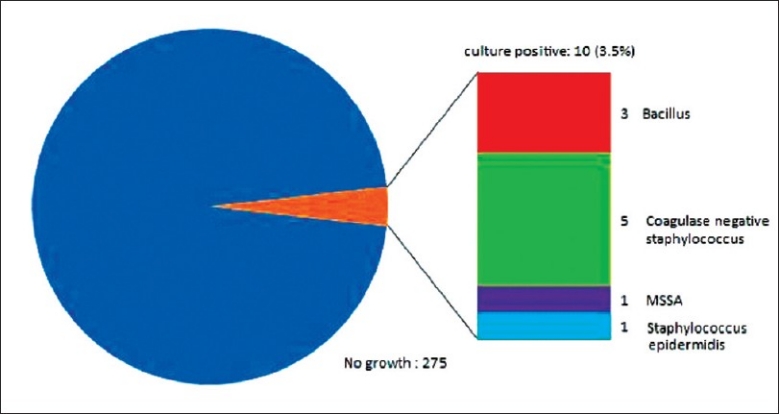
Ventriculostomy catheter tip culture

The intra-cranial end (drain tip) of the external ventricular drain would be sent for routine bacteria culture during operative revision or after removal under aseptic technique when the EVD were no longer required. A small number of ventricular drain tips were not saved for culture however, these include patients who died before the ventriculostomy were removed in which there were no clinical features suggesting a ventriculostomy-related infection. Only 10 cases (3.5%) had a positive culture of the EVD tip, the majority bacteria group was Coagulase negative staphylococcus with bacillus as the second most common. [Table 9, Figure 10].

**Table 9:** EVD tip culture

EVD tip culture	No. of cases
No growth	275
Total positive EVD culture	10
Coagulase negative staphylococcus (other than epidermidis)	5
Bacillus	3
MSSA	1
Staphylococcus epidermidis	1

#### Culture of the cerebral spinal fluid

Cerebral spinal fluid (CSF) samples were obtained for culture under aseptic techniques via the ventriculostomy catheter, intra-operatively during revision or in cases with ventriculostomy catheter already removed by lumbar puncture. CSF sampling via the ventriculostomy catheter were performed in cases with fever, raised serum white cell count, impaired mental status, duration of EVD longer than 5 days and manipulations which involved exposing the closed sterile external collection system for drained CSF (e.g. urokinase instillation, ICP monitor calibration, exchange of collection bag etc). CSF samples were obtained intra-operatively in all cases that required revision of ventriculostomy (irrespective of indication for revision) or in another neurosurgical operation when the ventriculostomy catheters were in situ. CSF samples were obtained by lumbar puncture after the removal of ventriculostomy catheter in cases which the routine bacterial culture of ventriculostomy catheter tip showed positive growth, suspicion of CNS infection or any other indication for lumbar punction (eg. Communicating hydrocephalus).

**Figure 11:**
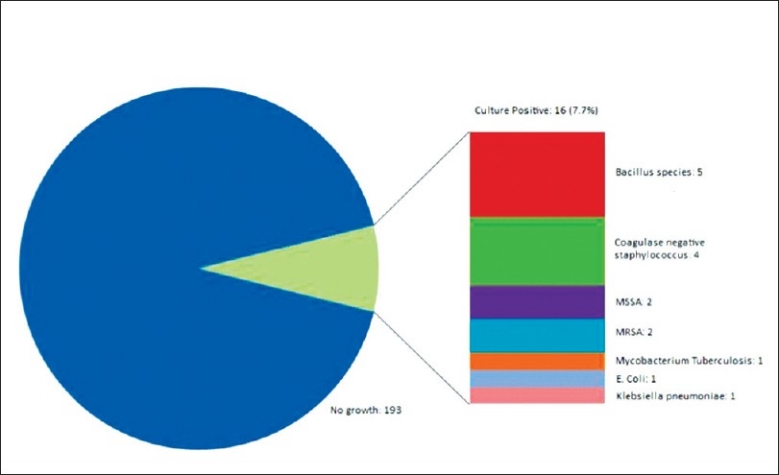
Culture

CSF culture was positive in 16 cases (7.7%), majority of the culture grew Bacillus species, Coagulase negative staphylococcus and staphylococcus aureus. [Table 10. Figure 11].

**Table 10:** CSF culture

CSF culture	No. of cases
No growth	193
Total CSF positive culture	16
Bacillus	5
Coagulase negative staphylococcus	4
MSSA	2
MRSA	2
MTB	1
*E. Coli*	1
Klebsiella pneumoniae	1

#### Bacillus outbreak

Bacillus infection outbreak occurred during the period from September to November of 2007 involving 4 cases of post-operative bacillus CNS infection. Extensive investigation found heavy growth of bacillus in the air ventilation system of the operation theatre and intensive care unit. Major disinfection was performed, and the infection rate returned to similar levels before the outbreak.[4]

### Ventriculostomy-related infection

A total of 23 patients had a positive culture result for CSF or EVD tip or both. Of these 23 patients, 8 patients were already diagnosed of meningitis before the procedure of ventriculostomy. Thus the remaining 15 patients had a positive culture after the procedure of ventriculostomy.

#### Definition of Ventriculostomy-related infection

Various studies had used different criteria for the definition for infection of ventriculostomy. Some studies simply defined infection by positive culture of the CSF or ventriculostomy catheter tip. Other studies took in some considerations including the clinical features of infection, biochemical or haematological laboratory test results and response to treatment.

For example:

Martinez[12] and Mayhall[13] used the term for infection as “nosocomial ventriculitis” which is defined as fever (>38.5 Degrees Celsius) more than 2 days after ventriculostomy catheter insertion, and a positive CSF culture.

Alan P. Lozier[11] proposed a classification to infection in case of ventriculostomy as “Contamination”, “Ventriculostomy colonization”, “Suspected ventriculostomy-related infection”, “Ventriculostomy-related infection” and “Ventriculitis“. The term of “Contamination” regarded as isolated positive CSF culture with normal CSF profile of protein, glucose and cell count. “Ventriculostomy colonization” being regarded as cases with repeated positive bacterial culture but no clinical symptoms of infection and normal CSF profile of protein, glucose and cell count.

Harrop JS[6] defined ventriculostomy infection as 2 positive CSF cultures from ventriculostomy catheters with a concurrent increase in cerebrospinal fluid white blood cell count.

GKC Wong[16] Defined infection as a positive CSF culture or fever in the absence of other causes and either a raised white cell count, raised protein, decreased glucose in CSF, or organisms visible on CSF Gram stain.

Using the various definitions of infection in the above-mentioned example with regards to the 15 cases of positive cultures in this review (pre-existing meningitis excluded) [Table 11].

**Table 11:** Definition comparison

Definition comparison		
Consider Literature	Case of Ventriculostomy Infection according to considered literature (Infection rate)	Case of exclusion reason
Martinez and Mayhall	12 (3.57)	3 case did not have fever
Alan P. Lozier	9 (2.68)	6 cases of contamination (isolated culture positive with normal CSF glucose, protein and colour)
Harrop JS	9 (2.68)	6 cases had insolated culture postive (repeated CSF culture negative)
G K W Wong	9 (2.68)	3 cases had fever and positive culture but normal CSF cell count, glucose and protein.
		3 cases had positive culture but no fever and normal CSF cell count, glucose and protein.

The infection rate varied in different criteria in definition of ventriculostomy-related infection, this difference is not statistically insignificant.

In this literature, we aim to use a definition of ventriculostomy-related infection with consideration of a positive culture together with of the parameters similar to the above-mentioned definitions of ventriculostomy-related infection. Thus we had decided on a less stringent criteria and defined ventriculostomy-related infection as a positive bacteria culture together with any two of the below mentioned individual factors from different subgroups.

Factors considered according to subgroups:

1. Clinical features: fever, nuchal rigidity, photophobia, seizures, alteration of conscious level or turbid sampled CSF appearance.
2. Biochemical test results: increase of CSF protein level, decrease of CSF glucose level or increase of serum inflammatory markers of C-reactive protein level.
3. Haematological test results: raised of CSF or blood white cell count.
4. Exclusion of other foci of infection: absent clinical features related to other sites of infection and insignificant culture from other possibility infected systems.

**Figure 12:**
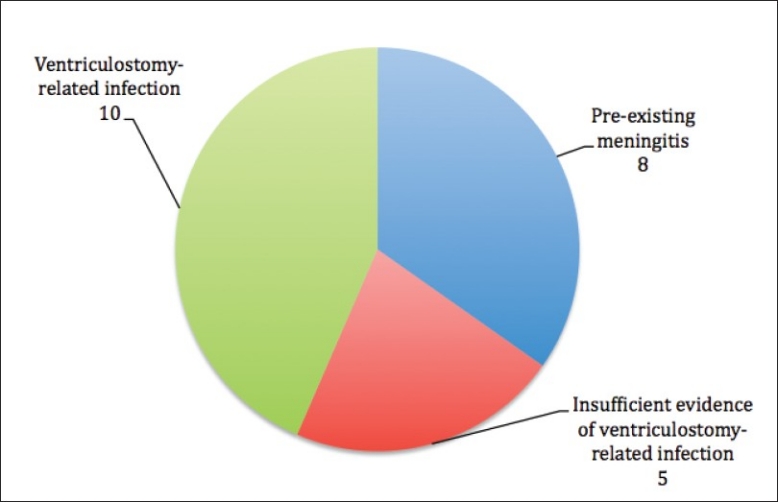
Breakdown of culture positive cases

From this definition, 10 cases (13 procedures of ventriculostomy) were identified as ventriculostomy-related infection. Thus the infection rate in this review is 2.98% (10/336) per case and 3.53% (13/368) per ventriculostomy procedure. [Figure 12].

### Bacterial culture of ventriculostomy related infection

**Figure 13:**
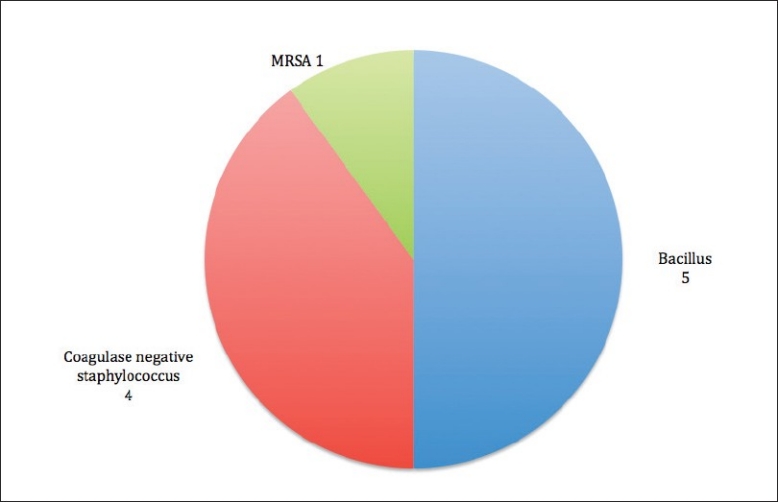
Bacteria for culture positive cases

Of the 10 cases of ventriculostomy-related infection, 5 cases involved bacterial culture of bacillus species all resistant to penicillin or methicllin. The 4 cases grew a bacterial culture of coagulase negative staphylococcus resistant to methicillin. 1 case grew methicillin resistant staphylococcus aureus. All were sensitive to vancomycin and gentamicin. 7 cases were treated with a course of intravenous vancomycin according to sensitivity. Intravenous gentamicin was given to 4 of the cases[9] and intraventricular irrigation with amikicin via the ventriculosotmy had been used in 3 of the cases.[15] [Figure 13].

### Duration of EVD for ventriculostomy related infection

The mean duration of the ventriculostomy in the 10 cases of ventriculostomy-related infection is 9 days.

4 out of the 10 cases of ventriculostomy related infection had duration of EVD above overall the average duration of 7.39 days in all the cases in this review. The difference between mean duration of EVD in ventriculostomy-related infection and non-infected case is statistically insignificant. Unpair two-tailed t test: *P*= 0.4135. [Table 12 and 13].

**Table 12:** Duration of infection cases

Duration of infection cases	Duration of EVD (days)
Mean	9
Standard deviation	± 7.10
Median	6
Shortest	2
Longest	23

**Table 13:** Ventriculostomy duration. (Infection vs no infection)

Ventriculostomy duration. (Infection vs no infection)	No. of cases	Mean duration of EVD (days)	Standard deviation (days)
Infection	10	9	7.1
Overall	336	7.39	6.1

### Revision of ventriculostomy and infection

27 cases in this review had revision, and among these cases, 2 (7.4%) were complicated with ventriculostomy-related infection. Compared to the cases without revision complicated by infection (8 cases, 2.59%), the difference is statistically insignificant with a Fisher’s exact test two-tailed *P*=0.1876 [Table 14].

**Table 14:** Revision (Infection vs no infection)

Revision (Infection vs no infection)	Revision	No revision	Total
Infection	2	8	10
No infection	25	301	326
Total	27	309	336
Percentage of infection	7.4%	2.59%	

### Urokinase instillation and infection

42 cases involved intraventricular urokinase instillation via the ventriculostomy,[10] only 1 case was complicated by ventriculostomy-related infection. The infection rate compared between the cases with urokinase instillation (2.38%) and with no urokinase instillation (3.06%) had no statistical significance with a Fisher’s exact test *P*-value of 1.000 [Table 15].

**Table 15:** Urokinase (Infection vs no infection)

Urokinase (Infection vs no infection)	Urokinase	No urokinase	Total
Infection	1	9	10
No infection	41	285	326
Total	42	294	336
Percentage of infection	2.38%	3.06%	

### Haemorrhage and infection

7 out of 10 cases of ventriculostomy-related infection in this study presented with haemorrhagic events involving 5 cases subarachnoid haemorrhages (SAH) and 2 cases intra-cranial haemorrhage. The 2 cases involved head trauma with resulting in acute epidural haemorrhage, acute subdural haematoma and multiple contusion. The infection rate of haemorrhagic event was 3.24% and the rate of haemorrhage not as primary event was 2.50%, statistically insignificance (Fisher’s Exact test *P*=1.000) [Table 16].

**Table 16:** Hemorrhage (Infection vs no infection)

Hemorrhage (Infection vs no infection)	Hemorrhage as primary presentation	Hemorrhage not as primary presentation	Total
Infection	7	3	10
No infection	209	117	326
Total	216	120	336
Percentage of infection	3.24	2.50	

### Multiple catheter insertions and infection

There were 5 cases in this review involved a duration in which more than one ventriculostomy catheter were present in the patient at any time. 1 of these 5 cases was complicated by ventriculostomy-related infection. Fisher’s exact two-tailed test *P*=0.141 (Statistically insignificant) [Table 17].

**Table 17:** Multiple catheter (Infection vs no infection)

Multiple catheter (Infection vs no infection)	Multiple catheter	Single catheter	Total
Infection	1	9	326
No infection	4	322	326
Total	5	331	336
Percent of infection	20	2.72	

### Summary of findings

Out of 336 cases of ventriculostomy, 10 cases were diagnosed with ventriculostomy-related infection. The infection rate per case is 2.98% (10/336) and infection rate per ventriculostomy 3.53% (13/368).

Various factors including duration of ventriculostomy, revision, urokinase instillation, haemorrhage and multiple catheterization were considered for the association with ventriculostomy-related infection. These factors are all statistically insignificant in this review.

## DISCUSSION

The ventriculostomy related infection rate in this review was 2.98% (only 10 out of 336 cases). This infection rate is lower compared to a majority of literatures. It is important to note however, the definition of ventriculostomy-related infection in the varied among studies. For example, an international literature review (Ventriculostomy-related Infections: A Critical Review of the Literature. Alan P. Lozier, Robert R. Sciacca, Mario F. Romagnoli, E. Sander Connolly, Jr. Neurosurgery Vol. 51, number 1, July2002) showed that the composite infection rate of all the studies in the review as 8.8% (2.13% to 21.95%). However, most of the studies defined infection as a positive culture result from the cerebrospinal fluid regardless of the clinical features, relevant laboratory test results and pre-existing meningitis. In order to compare our results to this composite infection rate, we should first compare with all our cases with positive culture of CSF or EVD tip which is 23 cases and 6.8% per patient and 6.25% per ventriculostomy. Statistically insignificant compared to composite international data [Table 18].

**Table 18:** Internation data compare

Internation data compare	International composite[11]	PYNEH
Culture positive	463	23
No. of patients	5261	336
No. of EVD	5733	368
Percentage of positive culture/patient	8.8% (463/5261)	6.8% (23/336)
Fisher’s Exact test	*P*=0.31	
Percentage of positive culture/EVD	8.08%	6.25% (23/336)
Fisher exact test: *P*=0.23		

### *Actual* ventriculostomy related infection

In the above mentioned literature review, there were a number of studies that defined ventriculostomy-related infection with regard to clinical features, laboratory test results and pre-existing meningitis. These studies were individually compared to this review of our hospital. The table below was obtained from the literature: Ventriculostomy-related Infections: A Critical Review of the Literature. Alan P. Lozier, Robert R. Sciacca, Mario F. Romagnoli, E. Sander Connolly, Jr. Neurosurgery Vol. 51, number 1, July2002 [Table 19].

**Table 19:** Data comparison with consideration of clinical feature

Series.	Patients	EVD	Positive culture	Rate per patient (%)	Rate per EVD (%)
Sundbarg *et al*.	540	540	54	10	10
Mayhall *et al*.	172	213	19	11.05	8.92
Schultz *et al*.	78	94	16	20.51	17.02
Holloway *et al*.	584	712	61	10.45	8.57
Lyke *et al*.	157	196	11	5.61	7.01

Sundbarg *et al*. classified positive CSF culture as a definite VRI if it was associated with CSF pleocytosis (defined as at least 11 leukocytes/mm^3^ with 50% or more polymorphonuclear neutrophils) and clinical symptoms that could not be attributed to causes other than ventriculitis. Several authors excluded patients with positive CSF cultures from the ventriculitis corhort on the ground that their infections were not primarily catheter related. The most widely accepted of such criteria required an initial sterile CSF culture obtained at the time of EVD insertion. Patients with positive CSF cultures at EVD insertion were diagnosed with preexisiting meningitis.

The results in this study were compared the statistics from the individual studies involved in the above mentioned literature review that included consideration of clinical features and pre-existing meningitis. The infection rate in our result was significantly lower compared to 4 out of 5 of the studies except for that of Lyke *et al*, (*P*=0.2798) which determined ventriculostomy-related infection as a positive bacterial culture after ruling out pre-existing meningitis but without considerations of clinical features. [Table 20].

**Table 20:** Compare international data

CSF culture positive (rate/patient)	Simple size	Culture positive (pre-existing meningitis excluded)	Culture negative or pre-existing meningitis	% of positive culture (pre-existing meningitis excluded)	Fisher’s exact test (compared to data of this study) *P* value	Culture positive and correlated clinical and laboratory information	Not ventriculostomy related infection	% of infection with clinical correlation	Fisher’s exact test (compared to data of this study) *P* value
PYNEH (this study)	336	15	321	4.46		10	326	2.98	
Mayhall *et al*. 1984	172	19	153	11.05	0.0078				
Schultz *et al*. 1993	94	16	78	20.51	0.0001				
Holloway *et al*. 1996	584	61	523	10.45	0.0012				
Lyke *et al*.	157	11	146	5.61	0.2798				
Sundbarg *et al*. 1988	540					54	486	10	0.0001

2 literatures, which were not included in the above-mentioned review,[11] did record a much lower infection rate. These two literatures, one by Friedman WA and Vries JK[5] and another by Bader, Littlejohns and Palmer[3] had stated an infection rate of 0%. A previous retrospective review of our centre for a 5-year-period prior the period of this study also demonstrated a low ventriculostomy-related infection rate of 0.92% (4/436).[1] The common feature among these literatures with low rate of infection was a very meticulous peri-operative management and strict nursing care aimed at reducing ventriculostomy-related infection. Bader, Littlejohns and Palmer[3] had a number of suggestions for methods to reduce infection.

At the author’s centre, a number of peri-operative management and nursing practices were observed to prevent infection of ventriculostomy (listed in the table below) [Table 21].

**Table 21:** Practice recommendation

Practice recommendation	Medical and operative management	Nursing care
Pre-operative and intra-operative	Peri-operative intravenous antibiotics Insertion of ventriculostomy only in the operative theatre Aseptic technique during entire operation with surgeons scrubbed and sterile gowned Scalp disinfected with 3 types of disinfectants (in sequel according to order of dis-infective power) before draped with sterile cloth If other neurosurgical procedures were performed in the same session, a separate skin incision and hole burr was used for insertion of EVD whenever possible Free drainage to room-air minimised upon immediate insertion of EVD to reduce pneumocephalus Percutanous tunnelling of at least 3cm before connection to external drainage system Catheter anchored to skin at site of percutanous tunnel exit	Patient’s head shaved for adequate exposure and reduce contamination from patient’s hair Aseptic technique during operative EVD insertion (nurse scrubbed and sterile gowned) with adequate sterile field. Sterile external drainage system assembled intra-operatively within the sterile field (by a gowned personal) The sterile ICP monitoring device was assemble in the external drainage system without direct contact with operative site External drainage system wrapped with anti-bacterial gel on sterile gauze and enclosed with water-proof material at all connection points Anti-septic gel applied to operative wound upon closure then dressed with sterile dressing
Post-operative (period which the catheter was in situ)	Post-operative care in ICU or HDU until ventriculostomy removed Patient monitored for symptoms or signs indicative of infection CSF and blood sampling at the slightest suspicion of infection Treatment of infection commenced when clinical features and laboratory test results suggest infection Revision of ventriculostomy NOT routinely performed unless clinically indicated Ventriculostomy removed as soon as the purpose of ventriculostomy had been fulfilled Ventriculostomy removal under strict aseptic technique and catheter tip routinely saved for bacterial culture	External drainage system maintained as a close system except during manipulation All manipulation of external drainage system keep to a minimal Aseptic technique (personal scrubbed and sterile gowned and establishment of a sterile field) when manipulation was required Sterile collection bag for CSF changed every once a week Upon manipulation of external drainage system CSF samplings from external system were performed

## CONCLUSION

The infection rate of ventriculostomy at our department in the previous 4 years has been low (2.98%). No factors considered in this study showed a significant associated to infection. This overall result is comparable or more ideal compared to most other international studies.

A collective effort of meticulous peri-operative management and stringent nursing care had been proven to successfully decrease the incidence of ventriculostomy-related infection in the author’s centre and other centers that recorded a low infection rate.
